# 18-Crown-6 and Dibenzo-18-crown-6 Assisted Extraction of Cesium from Water into Room Temperature Ionic Liquids and Its Correlation with Stability Constants for Cesium Complexes

**DOI:** 10.3390/molecules14125001

**Published:** 2009-12-02

**Authors:** Andrey G. Vendilo, Dmitry I. Djigailo, Svetlana V. Smirnova, Irina I. Torocheshnikova, Konstantin I. Popov, Vladimir G. Krasovsky, Igor V. Pletnev

**Affiliations:** 1 State Research Institute of Reagents and High Purity Substances (IREA), Bogorodskii val 3, 107258, Moscow, Russia; E-Mail: vendilo@ekos-1.ru (A.G.V.); 2 Department of Chemistry, M.V. Lomonosov Moscow State University, 1-3 Leninskiye gory, 119992, Moscow, Russia; E-Mails: djigailo@yandex.ru (D.I.D.), smirnova@analyt.chem.msu.ru (S.V.S.), dis@analyt.chem.msu.ru (I.I.T.); 3 Department of Physical and Colloid Chemistry, Moscow State University of Food Technologies, Volokolamskoye Sh. 11, 125080, Moscow, Russia; E-Mail: ki-popov@mtu-net.ru (K.I.P.); 4 Zelinsky Institute of Organic Chemistry RAS, Leninsky pr. 47, 119991, Moscow, Russia; E-Mail: miyusha@mail.ru (V.G.K.)

**Keywords:** ionic liquids, crown ethers, cesium extraction, stability constants

## Abstract

The pH-profiles of the extraction of Cs^+^ into four conventional (1-butyl-3- methylimidazolium hexafluorophosphate and bis[trifluoromethyl)sulphonyl]imides of 1- butyl-3-methylimidazolium, 1-hexyl-3-methylimidazolium, and 1-(2-ethylhexyl)-3-methylimidazolium) and two novel (trioctylmethylammonium salicylate and tetrahexyl-ammonium dihexylsulfosuccinate) room temperature ionic liquids have been determined both in the absence and in the presence of crown ether (18-crown-6 or dibenzo-18-crown-6). The pH-profiles of distribution ratio of crown ethers have been established in the same conditions. The relationship of cesium extraction efficiency both with the stability of its complexes with crown ethers and crown ethers’ distribution ratio has been clarified.

## 1. Introduction

Hydrophobic crown ethers are applied as extractants in the separation and recovery of ^137^Cs and ^90^Sr radioactive isotopes from high-level nuclear waste solutions, containing high concentrations of acids and salts, into molecular solvents [1,2,3,4,5]. Room-temperature ionic liquids (RTILs) are attracting increasing attention in solvent extraction processes due to important advantages over conventional organic diluents such as negligible vapor pressure, low flammability, moisture stability, unusual extraction properties and possibility to eliminate aqueous phase acidification [6,7,8,9,10,11,12,13]. It was demonstrated that extraction efficiency of RTIL can be modulated by using complexing agents, for example, crown ethers. Dai *et al.* [8] discovered that highly efficient extraction of strontium ions can be achieved when dicyclohexane-18-crown-6 is combined with RTILs. Rogers *et al.* [9] and Bartsch *et al.* [12] reported the extraction of various alkali metal ions with crown ethers in RTILs. Visser and Rogers demonstrated that octyl(phenyl)-*N,N*-diisobutylcarbamoylmethylphosphine oxide dissolved in RTILs enhances the extractability of lanthanides and actinides in comparison to conventional organic solvents [13]. The extraction of silver ions was found to be greatly enhanced by a combined application of RTIL and calix[4]arene compared to that of chloroform [14]. In addition, the task-specific RTILs with complexing functionality built in the RTIL cation have been reported [15,16]. Recently the efficiency of chelate extraction of 3d-cations with 8-sulfonamidoquinoline [17], Pu(IV) with carbamoylmethylphosphine oxide [18] and uranyl ion with tributylphosphate (TBP) [19] from aqueous phase into RTILs was reported. The higher selectivity of dibenzo-18-crown-6 to K^+^ over Na^+^ in *N*-octadecylisoquinolinium tetrakis[3,5-bis(trifluoromethyl)phenyl]borate compared with that in molecular solvents suggests that RTIL provides a unique solvation environment for the complexation of crown ethers with the ions [20].

**Scheme 1:**
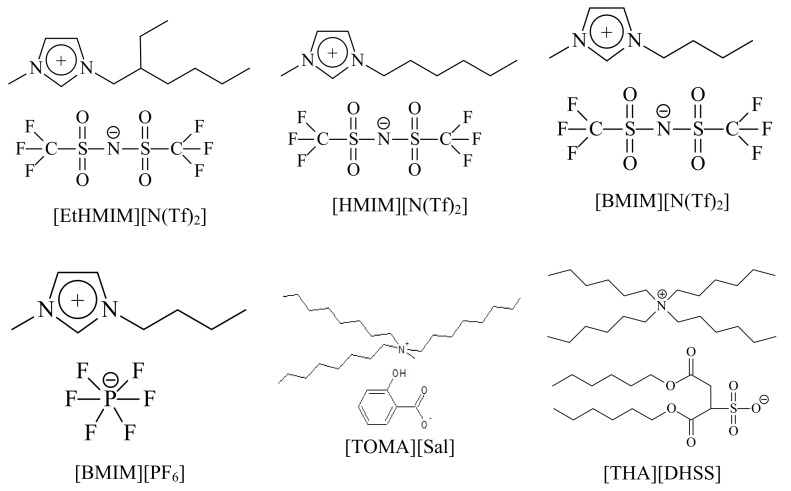
Structures of the RTILs studied.

Besides the issues of cation, ligand and complex solubility in water and in RTIL, the relative stabilities of complex formation in both phases are of significant importance for extraction selectivity. In our recent communication [21] we have reported on the thermodynamics of complex formation of cesium ions with 18-crown-6 (18C6, L) in six hydrophobic RTILs (see Scheme 1): trioctylmethylammonium salicylate ([TOMA][Sal]), tetrahexylammonium dihexylsulfosuccinate ([THA][DHSS]), 1-butyl-3-methylimidazolium hexafluorophosphate ([BMIM][PF_6_]), 1-butyl-3- methylimidazolium bis[trifluoromethyl)sulphonyl]imide ([BMIM][N(Tf)_2_]), 1-hexyl-3-methyl- imidazolium bis[trifluoromethyl)sulphonyl]imide ([HMIM][N(Tf)_2_]), and 1-(2-ethylhexyl)-3-methyl- imidazolium bis[trifluoromethyl)sulphonyl]imide ([EtHMIM][N(Tf)_2_]) by a ^133^Cs-NMR technique. The present work aims to study the extraction capability of cesium by the above mentioned RTILs in the presence of 18C6 and dibenzo-18-crown-6 (DB18C6, L) and to clarify its relationship with the stability of complexes formed in aqueous and organic phases. Another objective is to study the impact of crown ether’s nature on the extraction efficiency. For this reason the two crown ethers with similar cavity, but different hydrophobicity (18C6 and DB18C6) have been chosen.

## 2. Results and Discussion

The results of the extraction experiments are presented in Table 1, Table 2 and Table 3, where D_Cs_ refers to cesium distribution ratio for the extraction from aqueous phase into RTIL in the absence of crown ethers, D_Cs_^18C6^ as well as D_Cs_^DB18C6^ indicate the same in the presence of 18C6 and DB18C6, respectively, while D_18C6_^Cs^ and D_DB18C6_^Cs^ are distribution ratios for 18C6 and DB18C6 in the presence of cesium ions.

The RTILs studied demonstrate different extraction behavior towards cesium. [TOMA][Sal] and [THA][DHSS] appeared to be better solvents for this cation then water and provide positive logD_Cs_ values within the whole pH range, Table 1. This fact can be explained by the ability of salicylate and dihexylsulfosuccinate anions to interact with metal ion. Though such an interaction is not expected to be strong, worthy is mentioning that the bulk concentration of RTILs complexing anion is rather high: [Sal] = 1.68 and [DHSS] = 1.29 mol·dm^-3^. In contrast, conventional-anion RTILs [BMIM][PF_6_], [BMIM][N(Tf)_2_], [HMIM][N(Tf)_2_], and [EtHMIM][N(Tf)_2_] reveal negative logD_Cs_ values, which are in a good agreement with those found for [BMIM][PF_6_] earlier [12], Table 4. The cesium extraction ability of all RTILs, with the exception of [EtHMIM][N(Tf)_2_], slightly diminishes as pH decreases.

The situation becomes completely different when crown-ether molecules are introduced into RTIL/water biphasic systems. 18C6 significantly *decreases* cesium’s uptake by [THA][DHSS] but increases it for all other RTILs, Table 2. As far as we know, [THA][DHSS] is the first example of a negative influence of crown ether on a cation extraction into organic phase. Probably, the main reason is that crown-ether competes with a RTIL’s anion for cesium and, being better soluble in water, provides a partial transfer of the metal ion into an aqueous phase. This suggestion is indirectly supported by the low value of [Cs(18C6)]^+^ stability constant in [THA][DHSS] (log*K*_1_), Table 4, which appears to be even lower than in water.

**Figure 1:**
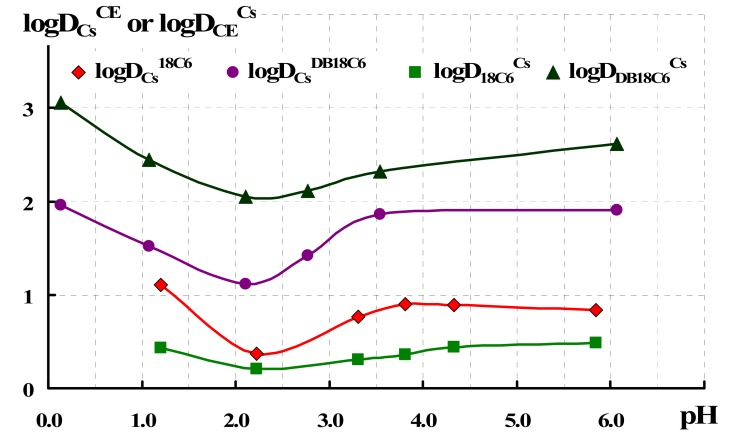
Distribution of cesium, 18C6, and DB18C6 vs. pH in [TOMA][Sal] based systems.

For DB18C6 an enhancement of cesium extraction is much more pronounced then for 18C6, Table 3. Notably, increasing HNO_3_ concentration in an aqueous phase decreases cesium’s recovery for all RTILs, but at very low pH both logD_Cs_^18C6^ and logD_Cs_^DB18C6^ start to increase. Thus, there appears a minimum at the curves logD_Cs_ = f(pH), in between pH 0 and pH 7 (Figure 1).

A similar effect has been observed in [9] for cesium distribution in [BMIM][PF_6_] in the presence of 4,4’-(5’)-di-(*tert*-butylcyclohexano)-18-crown-6. Notably, cesium recovery in strongly acidic media is usually lower than in neutral solutions (with the exception of systems 18C6/[THA][DHSS], 18C6/[TOMA][Sal], and DB18C6/[TOMA][Sal]). Thus, unlike crown ether solutions in conventional molecular solvents, such solutions in the studied RTILs do not need acidification to increase a recovery. Moreover, in most cases the addition of acid leads to the decrease of cesium recovery.

Worthy of mention is the fact that the extraction efficiency of solutions of CE in conventional solvents is often unsatisfactory even in strongly acidic media. According to Yakshin *et al.* [22], in the case of Cs^+^ (1 × 10^-3^ M) extraction from 2 M HNO_3_ aqueous solution into 0.1 M 18C6 in CHCl_3_ the value of D_Cs_ is extremely low (3 × 10^-3^; logD_Cs_ = -2.52). This value is lower than even the minimal values of D_Cs_ obtained in the case of our RTILs-based systems. As a rule, the extraction efficiency of the systems based on conventional solvents may be raised to the level of CE/RTIL-systems only by addition of a bulk hydrophobic counter-ion (such as picrate) to the aqueous phase. So, the logD_Cs_^18C6^ values for 1,2-dichloroethane in the presence of picrate in aqueous phase are of the same level that for some RTILs (e.g., [HMIM][N(Tf)_2_] and [TOMA][Sal], Table 4), while the hazardous properties of the former are incomparably higher.

The crown ether influence on cesium extraction by RTILs is evidently explained by a formation of rather stable complexes. Previously, we found [21] that Cs^+^ forms stable complexes [Cs(18C6)]^+^ in [TOMA][Sal], [THA][DHSS], [BMIM][PF_6_], [BMIM][N(Tf)_2_], [HMIM][N(Tf)_2_] and [EtHMIM][N(Tf)_2_], and the stability constants have been measured. Besides, in [BMIM][N(Tf)_2_], [HMIM][N(Tf)_2_], and [EtHMIM][N(Tf)_2_] the species [Cs(18C6)_2_]^+^ are also formed [21]. In an aqueous phase only [Cs(18C6)]^+^ species are registered [24].

Generally, when an aqueous phase of cesium nitrate is in equilibrium with a RTIL organic phase, containing crown ether L, the cesium distribution ratio D_Cs_^L^ (for such RTILs as [TOMA][Sal], [THA][DHSS] and [BMIM][PF_6_]), where only CsL species are formed is represented by a simple equation (1):

D_Cs_^L^ = ([Cs]^RTIL^ + [CsL]^RTIL^)/([Cs]^w^ + [CsL]^w^) (1)

where the superscripts “RTIL” and “w” denote organic and aqueous phase, respectively. When the total concentration [L]^t^ >> [Cs]^t^ and the stabilities of complexes in both phases are high enough (log*K*_1_^RTIL^ ≥ 2; log*K*_1_^w^ ≥ 2), then [Cs]^RTIL^ << [CsL]^RTIL^ and [Cs]^w^ << [CsL]^w^. In this case, the equilibrium concentrations of [Cs]^RTIL^ and [Cs]^w^ in (1) can be neglected. The equation (1) is therefore transformed into (2):

D_Cs_^L^ = [CsL]^RTIL^/[CsL]^w^ (2)

or:

D_Cs_^L^ = (*K*_1_^RTIL^[Cs]^RTIL^[L]^RTIL^)/(*K*_1_^w^[Cs]^w^[L]^w^). (3)

As far as [Cs]^RTIL^/[Cs]^w^ = D_Cs_ and [L]^RTIL^/[L]^w^ = D_L_:

D_Cs_^L^ = *K*_1_^RTIL^ D_Cs_D_L_/*K*_1_^w^ (4)

or:

logD_Cs_^L^ = log*K*_1_^RTIL^ + logD_Cs_ + logD_L_ − log*K*_1_^w^. (5)

**Figure 2:**
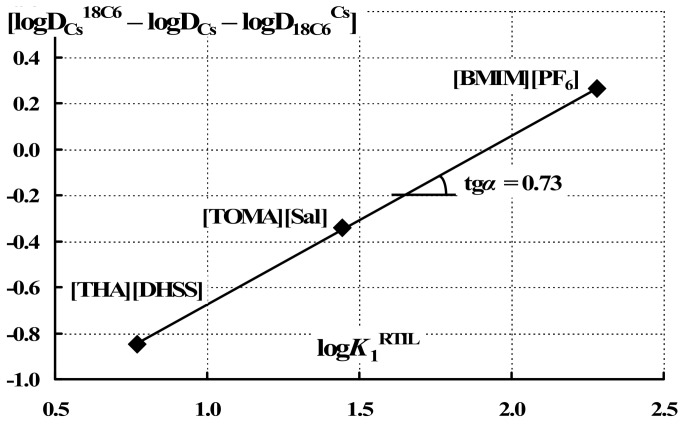
A plot of [logD_Cs_^18C6^ − logD_Cs_ − logD_18C6_^Cs^] vs. log*K*_1_^RTIL^ for [TOMA][Sal], [THA][DHSS] and [BMIM][PF_6_] at 22–25 °C.

Thus, the plot of (logD_Cs_^L^ − logD_Cs_ − logD_L_) versus log*K*_1_^RTIL^ was expected to be linear with a slope of 1. In our case the initially stated requirement log*K*_1_^RTIL^ ≥ 2; log*K*_1_^w^ ≥ 2 does not take place for some RTILs and for water. Indeed, log*K*_1_^w^ = 0.98 [24] and for the RTILs the following values of log*K*_1_^RTIL^ at 25 °C have been determined [21]: 1.45 (0.05) ([TOMA][Sal]); 0.77 (0.04) ([THA][DHSS]); 2.4 (0.2) ([BMIM][PF_6_]); 3.4 (0.5) ([BMIM][N(Tf)_2_]); 4.4 (0.1) ([HMIM][N(Tf)_2_]), and 3.4 (0.4) ([EtHMIM][N(Tf)_2_]). Nevertheless, the plot of (logD_Cs_^18C6^ − logD_Cs_ − logD_18C6_^Cs^) versus log*K*_1_^RTIL^ for [TOMA][Sal], [THA][DHSS], and [BMIM][PF_6_] represents a perfectly strait line with a slope 0.73 and R^2^ = 0.999, Figure 2, Table 4. In order to confirm the legitimacy of the derivation of the equation 4 we have determined the concentration profiles of D_Cs_ for all RTILs in the range C_w_^0^(Cs) = 2 × 10^−4^ − 5 × 10^−2^ M. In all cases D_Cs_ remains nearly constant at 2·10^−4^ ≤ C_w_^0^(Cs) ≤ 1.5·10^−3^ M (≤ 5·10^−3^ M − for [TOMA][Sal] and [THA][DHSS]). At the same time the maximal cesium recovery (in the case of 18C6) is observed for 18C6/[BMIM][N(Tf)_2_] at pH = 6.88 (Table 2), which corresponds to the residual free cesium concentration being equal to 2.9 × 10^−4^ M (*i.e.*, even in this case the ratio [free Cs in the organic phase]/[free Cs in the aqueous phase] is the same that in the case of Cs^+^-extraction from 1.5 × 10^−3^ M aqueous solution in the absence of crown ether in RTIL-phase).

**Figure 3:**
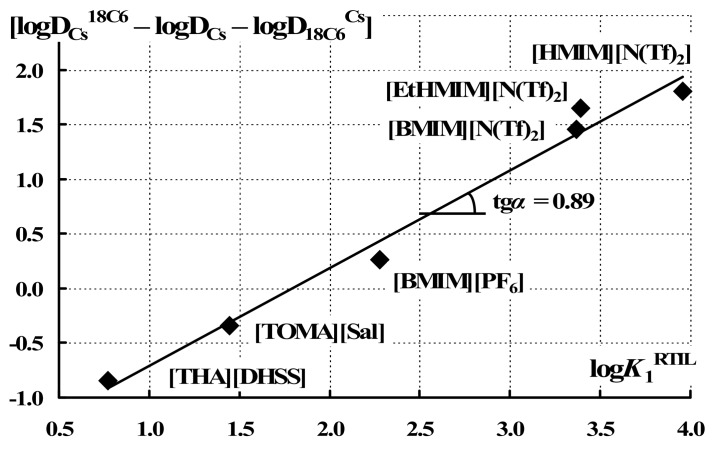
A plot of [logD_Cs_^18C6^ − logD_Cs_ − logD_18C6_^Cs^] vs. log*K*_1_^RTIL^ for [TOMA][Sal], [THA][DHSS], [BMIM][PF_6_], [BMIM][N(Tf)_2_], [HMIM][N(Tf)_2_], [EtHMIM][N(Tf)_2_] at 22–25 °C.

In the case of [BMIM][N(Tf)_2_], [HMIM][N(Tf)_2_], [EtHMIM][N(Tf)_2_] the equations (1), (2), (4) and (5) become more complicated due to formation of CsL_2_ complexes and the lack of such complexes in water. However, all six RTILs fit equation (5) with a slope 0.89 and a correlation coefficient R^2^ = 0.98, Figure 3. This can be explained by a domination of log*K*_1_^RTIL^ values over log*K*_2_^RTIL^ ones, Table 4. Another reason of low contribution of *K*_2_^RTIL^ to the extraction is in the absence of enough sufficient excess of crown re. cesium concentration. Anyway, the linear relationship of (logD_Cs_^18C6^ − logD_Cs_ − logD_18C6_^Cs^) and log*K*_1_^RTIL^ indicates clearly an importance of complex stability contribution for the extraction of cesium into hydrophobic RTILs.

Besides log*K*_1_^RTIL^, also a contribution of logD_L_ should be examined. Thus, along with cesium extraction we have studied the water/RTIL distribution of crown ethers, Table 2 and Table 3. 18C6 demonstrates similar affinity towards as the RTILs, so the conventional molecular solvents: 1,2-dichloroethane, chloroform and dichloromethane [5]. The detaining of DB18C6 in extracts is sufficiently higher for all RTILs then that of 18C6, Table 2 and Table 3. At the same time logD_DB18C6_^Cs^ values are one to two log unites lower then those found for benzene, 1,2-dichloroethane, chloroform and nitrobenzene [5].

**Figure 4:**
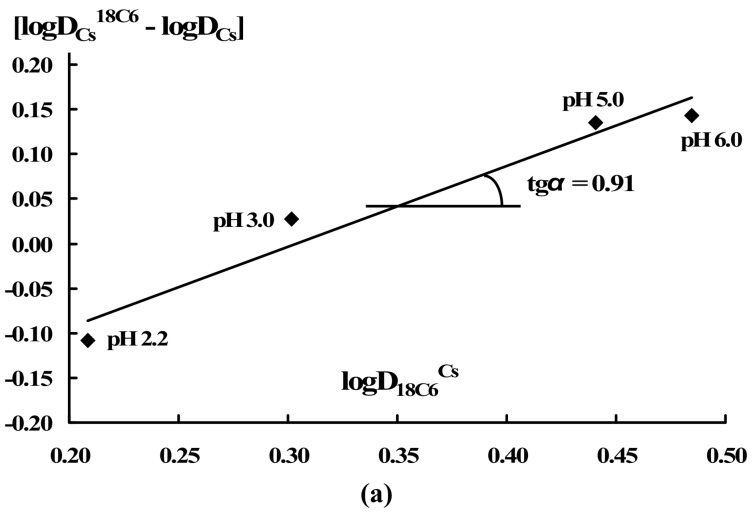

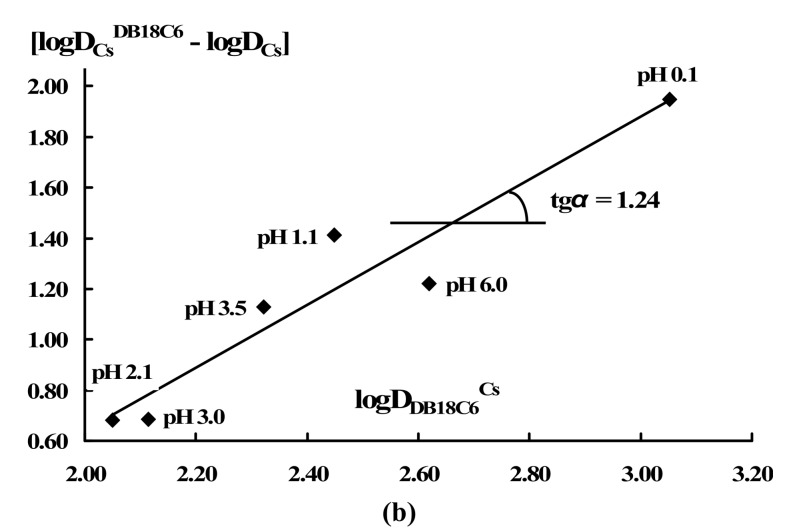
Plots of [logD_Cs_^CE^ − logD_Cs_] vs. logD_CE_^Cs^ for TOMAS at 22 °C. **(a)** CE = 18C6; **(b)** CE = DB18C6.

It should be noted that the variation of logD_Cs_^18C6^ and logD_Cs_^DB18C6^ with pH shows the same trend as the crown ether distribution between aqueous phase and RTIL, logD_18C6_^Cs^ and logD_DB18C6_^Cs^, Figure 1. A linear relationship between logD_18C6_^Cs^ and logD_Cs_^18C6^ (slope 0.91; R^2^ = 0.96) as well as between logD_DB18C6_^Cs^ and logD_Cs_^DB18C6^ (slope 1.24; R^2^ = 0.91) was found for TOMAS, Figure 4, indicating the dominating role of relative crown ether solubility in water and RTIL.

Analysis of the data presented in Table 4 for [BMIM][N(Tf)_2_], [HMIM][N(Tf)_2_], and [EtHMIM][N(Tf)_2_] indicates that the contribution of logD_18C6_^Cs^ into logD_Cs_^18C6^ prevails over log*K*^RTIL^ values. Indeed, the variation of logD_Cs_^18C6^ within this set of similar RTILs with the same anion definitely follows the changes in logD_18C6_^Cs^ values but not the stability constants variations.

A comparison of 18C6 and DB18C6 indicates a much higher extraction efficiency of the latter. The stability constants of cesium complexes with DB18C6 in the studied RTILs have not been measured yet, but for other than RTIL solvents they reveal 0.2 to 0.5 log units lower values than those for 18C6 [25]. However, bearing in mind that this is also valid for an aqueous solution, the difference in (log*K*_1_^RTIL^ − log*K*_1_^w^) in Equation (5) becomes negligible. Moreover, this unfavorable for extraction loss is excessively compensated by the fact that the values of logD_DB18C6_^Cs^ are 2 to 3 log units higher than logD_18C6_^Cs^ values.

## 3. Experimental

### 3.1. General

[HMIM][N(Tf)_2_] (Solvent Innovation; puriss. 99%), Bromothymol Blue (Baum-Lux, Russia; analytical reagent grade), KNO_3_ (analytical reagent grade), chloroform (Ekros, Russia), and absolute ethanol (analytical reagent grade) were used as received. Cesium nitrate (Aldrich, 99.99%) was dried for 6 hours at 70 °C, and then kept 24 h in a desiccator before use. Dibenzo-18-crown-6 (DB18C6, 99% purity) was synthesized by Dr. V.M. Polosin and analyzed by ^1^H-NMR. NMR revealed no other peaks than those corresponding to DB18C6. 18C6 (Aldrich, 99%) and DB18C6 have been melted at 70 and 180 °C, respectively, in a drying box for 3 h and then kept at 25 °C in a desiccator for 24 h to remove water before use. NMR spectra were recorded on a 500 MHz Bruker DRX500 instrument is the indicated solvents using TMS as reference.

### 3.2. Synthesis and characterization of the RTILs

*Trioctylmethylammonium salicylate.* Aliquat® 336 (~0.2 mol) was mixed with 30% excess of sodium salicylate in chloroform (200 mL). The mixture was shaken for 4 h and then rinsed 20 times with a large amount of distilled water, then the solvent was evaporated and the liquid residue was heated up to 100 °C under reduced pressure for 5 h. After cooling to room temperature a white solid matter was obtained with a density 0.943 g·cm^−3^; T_m_ = (32.8 ± 0.4) °C, T_f_ = (14 ± 2) °C. Yield: 90%. ^1^H-NMR (CDCl_3_) 0.88 (9 H), 1.24 (30 H), 1.59 (6 H), 3.19 (3 H), 3.27 (6 H), 7.20 (1 H), 7.92 ppm (1 H); ^13^C-NMR (DMSO-d_6_) 13.80 (C*CH_3_), 21.93 (C*H_2_CH_3_), 21.25, 25.68, 28.27, 28.32, 31.04 (various CH_2_CH_2_ fragments); 47.41 (CH_3_N); 60.50 (CH_2_N); 115.35, 115.62, 120.73, 129.73, 130,88 (aromatic C); 163.17 (COH); 171.00 ppm (COO). Analysis: found: C, 76.06; H, 11.64; N: 2.62; calc.: C, 76.49; H, 11.89; N: 2.62 (taking into consideration 2:1 mol/mol relation between methyltrioctyl- and methyltridecylammonium cations in Aliquat® 336). After equilibration with water at ambient temperature the solid product transforms into a slightly yellowish viscous liquid with 0.942 g·cm^−3^ density and a freezing point below −18 °C. Water content measured by Karl Fisher titration constituted 0.18% wt. (0.09 mol·dm^−3^) for a solid product and 4.83% wt. (2.52 mol·dm^−3^) for that equilibrated with water RTIL.

*Tetrahexylammonium dihexylsulfosuccinate* was synthesized according to [26] as a transparent viscous liquid (yield: 85%), analyzed by NMR, and then used without further purification. ^1^H-NMR (CDCl_3_) 0.88 (18 H), 1.33 (36 H), 1.55 (12 H), 3.2 (12 H), 4.1 (3 H) ppm; 13C-NMR (DMSO-d_6_) 13.64, 13.69, 13.71 (CH_3_); 20.91, 21.76 (C*H_2_CH_3_), 21.87, 24.82, 24.85, 25.34, 27,95, 27.99, 30.47, 30.75, 30.82 (CH_2_CH_2_ fragments); 34.01 (OOC*CH_2_CH-); 57.63 (CH_2_N); 63.89 (CH_2_O); 168.31, 170.97 COOR); Analysis: found: C, 66.67; H, 11.31; N: 1.97, S: 4.49; calc.: C, 66.71; H, 11.34; N: 1.94, S: 4.45. An absence of halogen-ions was proven by a AgNO_3_ test.

*1-Butyl-3-methylimidazolium hexafluorophosphate.* A 1-L, one-necked, round-bottomed flask was equipped with a magnetic stirrer and charged with 1-butyl-3-methylimidazolium chloride (367.2 g, 2.10 mol, 1 equiv.) and potassium hexafluorophosphate (387.3 g, 2.10 mol, 1 equiv.) in distilled water (700 mL). The reaction mixture was stirred at room temperature for 2 h, affording a two-phase system. The organic phase was separated and washed with water (5 × 40 mL) until the aqueous fraction observed to be free of chloride (AgNO_3_). Then dichloromethane (400 mL) was added. The dichloromethane solution was mixed with activated charcoal, stirred for 2 h, filtered and dried over anhydrous magnesium sulfate. After 1 h, the suspension was filtered and the volatile material was removed by rotary evaporation. The resulting colourless or light-yellow viscous liquid was dried under reduced pressure (0.5 mm Hg) at 70 °C for 12 h. Yield 462 g (77.3%). ^1^H-NMR (300 MHz, DMSO-d_6_): 0.91 (3H, t, NHCH_2_CH_2_CH_2_CH_3_), 1.27 (2H, m, NCH_2_CH_2_CH_2_CH_3_), 1.77 (2H, p, NCH_2_CH_2_CH_2_CH_3_), 3.85 (3H, s, NCH_3_), 4.16 (2H, t, NCH_2_CH_2_CH_2_CH_3_), 7.72 (2H, m, C(5)H, C(4)H), 9.09 (1H, s, C(2)H); Analysis: found: C, 33.97; H, 5.37; N, 9.95; F, 39.91; P, 10.78; calc.: C, 33.81; H, 5.32; N, 9.86; F, 40.11; P, 10.90.

*1-Butyl-3-methylimidazolium bis[trifluoromethyl)sulphonyl]imide.* A 500-mL, one-necked, round- bottomed flask was equipped with a magnetic stirrer and charged with lithium bis(trifluoromethylsulfonyl)imide (143.693 g, 0.5 mol, 1 equiv.) and 1-butyl-3-methylimidazolium chloride (87.25 g, 0.5 mol, 1 equiv.) dissolved in distilled water (80 mL). The reaction mixture was stirred at room temperature for 2 h affording a two-phase system. Then dichloromethane (200 mL) was added. The lower organic layer was separated and washed with distilled water (5 × 20 mL) until the aqueous fraction observed to be free of chloride (AgNO_3_). The dichloromethane solution was mixed with activated charcoal, stirred for 2 h, filtered and dried over anhydrous magnesium sulfate. After 1 h, the suspension was filtered and the volatile material was removed by rotary evaporation. The resulting colourless viscous liquid was dried under reduced pressure (0.5 mm Hg) at 70 °C for 12 h. Yield 162.6 g (77.5*%*). ^1^H-NMR (250 MHz, DMSO-d_6_): 0.89 (3H, t, NHCH_2_CH_2_CH_2_CH_3_), 1.25 (2H, m, NCH_2_CH_2_CH_2_CH_3_), 1.76 (2H, p, NCH_2_CH_2_CH_2_CH_3_), 3.84 (3H, s, NCH_3_), 4.15 (2H, t, NCH_2_CH_2_CH_2_CH_3_), 7.72 (2H, m, C(5)H, C(4)H), 9.09 (1H, s, C(2)H); Analysis: found: C, 28.67; H, 3.63; N, 9.94; F, 27.30; S, 15.23; O, 15.23; calc.: C, 28.64; H, 3.61; N, 10.02; F, 27.18; S, 15.29; O, 15.26.

*1-(2-Ethylhexyl)-3-methylimidazolium bis[trifluoromethyl)sulphonyl]imide* was kindly supplied by Dr. V.E. Baulin (A.N. Frumkin Institute of Physical Chemistry and Electrochemistry of RAS). ^1^H-NMR (200 MHz, CDCl_3_) 0.85 (m, 6H, 2CH_2_CH_3_), 1.25 (m, 8H, 4CH_2_), 1.75 (m, 1H, CH_2_CHCH_2_), 3.92 (s, 3H, CH_3_N), 4.00 (d, J=7.0 Hz, 2H, CH_2_N), 7.25(s, 1H_arom_), 7.36 (s, 1H_arom_), 8.65 (s, 1H_arom_); Analysis: found: C, 35.40; H, 4.85; F, 24.00; N: 8.81; S: 13.52; calc.: C, 35.36; H, 4.88; F, 23.97; N: 8.84, S: 13.49.

### 3.3. Extraction

For a sample preparation the weighted amount of solid CsNO_3_ was dissolved in a distilled water to obtain the initial solution of salt (1.5·10^-2^ mol·dm^-3^), which was further used for the preparation of the diluted samples. For adjusting pH values of aqueous phase, 0.01–3 M HNO_3_ (analytical reagent grade) was used.

Cesium extraction process in the absence of crown ethers was performed by adding of water- saturated RTIL samples (0.5 mL for [TOMA][Sal], [THA][DHSS], and [BMIM][PF_6_]; 1.0 mL for [BMIM][N(Tf)_2_], [EtHMIM][N(Tf)_2_] and 2.0 mL for [HMIM][N(Tf)_2_]) to 1.5 × 10^-3^ mol·dm^-3^ (2 × 10^-4^, 5 × 10^-4^, 5 × 10^-3^, 1 × 10^-2^, and 5 × 10^-2^ mol·dm^-3^ only at neutral pH, without addition of HNO_3_) CsNO_3_ aqueous solutions (5.0 mL for [TOMA][Sal], [THA][DHSS], and [BMIM][PF_6_]; 3.0 mL for [BMIM][N(Tf)_2_], [EtHMIM][N(Tf)_2_] and 2.0 mL for [HMIM][N(Tf)_2_]). The biphasic systems were shaken for 2 h (adequate time for equilibrium state establishment for all RTILs studied at room temperature, 22 °C). After centrifugation for 15 min aqueous and organic phases were separated. An aqueous phase was analyzed for equilibrium pH (pH-meter pH-410; combined glass microelectrode ESLK-13.7, Aquilon, Russia) and cesium content (flame photometry, FPA-2; Zagorsk Optical and Mechanical Plant, Russia). The recovery (R(Cs), *%*) and the distribution constant (D(Cs)) of cesium were found according to eqns. (6) and (7):

R(Cs), % = [1 – C_w_(Cs)/C_w_^0^(Cs)]·100%, (6)

D(Cs) = C_o_(Cs)/C_w_(Cs) = [V_w_/V_o_][R(Cs), %]/[100 – R(Cs), %], (7)

where C_w_^0^(Cs) and C_w_(Cs) are the initial and equilibrium concentrations of Cs^+^ in an aqueous phase, respectively, V_w_ и V_o_ correspond to the total volumes of aqueous and organic phase, respectively (mL). The ratio V_w_:V_o_ was 10 ([TOMA][Sal], [THA][DHSS], and [BMIM][N(Tf)_2_]), 3 ([BMIM][PF_6_], [EtHMIM][N(Tf)_2_]) and 1 ([HMIM][N(Tf)_2_]). To compensate the matrix effects the calibration standards were prepared by adding standard solutions of Cs^+^ to the aqueous solutions pre-equilibrated with the corresponding RTILs. Otherwise the error increased sufficiently.

For cesium extraction study in the presence of crown ethers, the precise amounts of 18C6 or DB18C6 were dissolved in the RTILs saturated by a distilled water. Initial concentration of 18C6, C(18C6)_0_ was 1.5 × 10^-1^ mol·dm^-3^ for all RTILs studied. The same concentration was used for DB18C6 in [BMIM][N(Tf)_2_]. Due to the solubility problems, the content of DB18C6 in [TOMA][Sal] and [THA][DHSS] was lower (C(DB18C6)_0_ = 5 × 10^-2^ mol·dm^-3^). This concentration was obtained after heating of these RTILs with the amounts of DB18C6 at 80–90 °C. Thus, these solutions are likely to be oversaturated, although no precipitation after cooling to room temperature was observed.

To provide an equal ratio C(CE)_0_:C(Cs^+^)_0_ = 100 and *ν*(CE)_0_:*ν*(Cs^+^)_0_ = 10 for all RTILs studied, the initial concentration of Cs^+^ in aqueous phases contacting with [TOMA][Sal] and [THA][DHSS] was adjusted to C(Cs^+^)_0_ = 5 × 10^-4^ mol·dm^-3^. The volume ratio V_w_:V_o_ = 10 was used for all 9 extraction systems studied (0.5 mL crown ether solution in RTIL and 5.0 mL of cesium nitrate aqueous solution with various pH). The aqueous solutions pre-equilibrated with the corresponding CE/RTIL systems were used for preparation of the calibration standards. All other experimental conditions were the same as for experiments run without crown ether.

Distribution of DB18C6 was studied in a parallel way within the same experiments on cesium extraction. The samples taken from phases of DB18C6/[TOMA][Sal], DB18C6/[THA][DHSS], and DB18C6/[BMIM][N(Tf)_2_] were dissolved in an absolute ethanol taken in vol/vol ratio 1000 (for DB18C6/[BMIM][N(Tf)_2_]) and 300 (for DB18C6/[TOMA][Sal] and DB18C6/[THA][DHSS]). Then the resulting solutions were analyzed spectrophotometrically (U-2900 UV-Vis Recording Spectrophotometer, Hitachi, Japan; 1 cm quartz cells) for DB18C6 content using crown ether absorption band λ_max_ = 274 nm (ε_max_ = 5,000 ÷ 5,500) and the reference solutions of the corresponding RTILs in ethanol with the same dilution as for DB18C6/RTILs. Detaining (R(CE), %) for DB18C6 has been calculated using equation (8):

R(Cs), % = [C_o_(Cs)/C_o_^0^(Cs)]·100%, (8)

where C_o_^0^(CE) and C_o_(CE) denote initial and equilibrium concentrations of crown ether in organic phase (extract), respectively; while distribution constant (D(CE)) − using equation (7).

Distribution of 18C6 was controlled by the method described elsewhere [27]. Bromothymol Blue (H-BTB) and KNO_3_ were dissolved in an aliquot taken from an aqueous phase (to adjust 1.2 × 10^-2^ mol·dm^-3^ concentration of both of them). Then the extraction of the complex [K(18C6)+·BTB–] into CHCl_3_ was performed with 2 h contact time and phases volume ratio 1:1; pH of an aqueous phase was kept constant (pH = 6.9) by phosphate buffer for all solutions other then neutral. Then the system was centrifuged for 15 min, an organic phase was separated and analyzed spectrophotometrically (λ_max_ = 400 ÷ 450 nm, ε_max_ = 1,500 ÷ 2,000; the chloroform extract obtained in the blank experiment was used as the reference solution). Detaining (R(CE), %) and distribution ratio (D(CE)) were calculated by the eqns., completely analogous to (1) and (2), taking into consideration that C_o_(CE) = C_o_^0^(CE) − 10·C_w_(CE), where C_w_(CE) denotes an equilibrium concentration of crown ether in aqueous phase.

## 4. Conclusions

The data presented reveals that several ionic liquids are suitable for extraction of cesium in the presence of crown ethers. The liquids which show a higher retention of a crown ether and a higher stability of the complexes demonstrate better capabilities to extract Cs^+^. The former factor dominates evidently over the latter one. Among the crowns the ligand of a choice is the one that demonstrates higher hydrophobicity, and, therefore, a higher detaining in extract, e.g., DB18C6.

## Figures and Tables

**Table 1:** Distribution ratios of cesium (C_w_^0^(Cs) = 1.5 × 10^-3^ mol·dm^-3^) between RTIL/aqueous phases in the absence of crown ether as a function of pH at 22 °C.

RTIL	Vo, mL	Vw, mL	Aqueous pH	logD_Cs_
[THA][DHSS]	0.5	5.0	5.94	1.21
			3.36	1.11
			1.59	0.84
[TOMA][Sal]	0.5	5.0	5.98	0.69
			4.95	0.76
			3.01	0.74
			1.13	0.10
[BMIM][PF_6_]	0.5	5.0	5.90	-0.59
			3.33	-0.82
			1.25	-0.80
[BMIM][N(Tf)_2_]	1.0	3.0	6.44	-0.67
			3.35	-1.24
			1.25	-1.31
[EtHMIM][N(Tf)_2_]	1.0	3.0	6.34	-0.81
			2.09	-0.62
[HMIM][N(Tf)_2_]	2.0	2.0	5.82	-1.24
			2.84	-1.36
			0.93	-2.21

**Table 2:** Distribution ratios of cesium [C_w_^0^(Cs) = 1.5 × 10^-3^ mol·dm^-3^] and 18C6 [C_o_^0^(18C6) = 1.5 × 10^-1^ mol·dm^-3^] in the presence of each other between RTIL/aqueous phases as a function of pH at 22 °C (V_o_ = 0.5 mL; V_w_ = 5.0 mL).

RTIL	Aqueous pH	logD_Cs_^18C6^	logD_18C6_^Cs^
[THA][DHSS]	5.92	0.24	-0.12
	4.30	0.23	-0.16
	3.86	0.03	-0.41
	3.39	-0.38	-0.50
	2.48	0.57	-0.15
	1.60	0.75	-0.01
[TOMA][Sal]	5.84	0.83	0.48
	4.33	0.89	0.44
	3.81	0.90	0.36
	3.31	0.77	0.30
	2.22	0.36	0.21
	1.20	1.11	0.43
[BMIM][PF_6_]	5.84	-0.20	0.13
	4.33	-0.45	0.08
	3.81	-0.62	0.06
	3.31	-1.27	0.00
	2.22	-0.74	0.00
	1.20	-0.66	0.08
[BMIM][N(Tf)_2_]	6.88	1.56	0.77
	5.60	1.43	0.65
	3.56	-0.26	0.50
	2.26	-0.27	0.08
	1.27	-0.23	0.23
[EtHMIM][N(Tf)_2_]	6.50	0.56	-0.27
	2.15	0.30	-0.45
[HMIM][N(Tf)_2_]	5.60	0.82	0.25
	4.10	0.91	0.17
	2.96	0.61	0.06
	1.40	-0.22	0.08
	0.90	-0.33	0.27

**Table 3:** Distribution ratios of cesium and DB18C6 in the presence of each other between RTIL/aqueous phases as a function of pH at 22 °C (V_o_ = 0.5 mL; V_w_ = 5.0 mL).

RTIL	C_w_^0^(Cs), mol·dm^-3^	C_o_^0^(18C6), mol·dm^-3^	Aqueous pH	logD_Cs_^DB18C6^	logD_DB18C6_^Cs^
[THA][DHSS]	5.0·10-4	5.0·10-2	6.03	1.56	2.68
			2.85	1.27	2.41
			2.45	0.89	2.20
			1.89	0.74	2.15
			1.06	0.86	2.30
			0.14	1.02	2.68
[TOMA][Sal]	5.0·10-4	5.0·10-2	6.07	1.91	2.62
			3.54	1.86	2.32
			2.77	1.42	2.11
			2.11	1.12	2.05
			1.08	1.52	2.45
			0.13	1.96	3.05
[BMIM][N(Tf)_2_]	1.5·10^-3^	1.5·10-1	6.93	2.28	2.89
			5.09	2.06	2.71
			3.16	0.14	2.57
			1.92	-0.47	2.63
			0.38	-0.60	2.94

**Table 4:** Stability Constants of Cesium Complex Formation with 18C6 in RTIL and Extraction Efficiency from Water at 22 °C and pH 5 – 7^a^.

Solvent	logK_1_	logK_2_	logD_Cs_	logD_Cs_^18C6^	logD_18C6_^Cs^	log[D_Cs_^18C6^/(DCsD_18C6_^Cs^)]
[HMIM][N(Tf)_2_]	4.4	1.13	−1.24	0.82	0.25	1.81
[EtHMIM][N(Tf)_2_]	3.4	1.16	−0.81	0.56	−0.27	1.64
[BMIM][N(Tf)_2_]	3.4	1.29	−0.67	1.56	0.77	1.46
[BMIM][PF_6_]	2.3		−0.59 −0.59^b^ −1.17^c^	−0.20 −0.52^d^	0.13 0.15^e^	0.26
[TOMA][Sal]	1.45		0.69	0.83	0.49	−0.38
[THA][DHSS]	0.77		1.21	0.25	−0.12	−0.84
1,2-dichloroethane	7.98^f^	2.58^f^	−7.7^g^	0.8^h^	0.03^i^	6.93

^a^ Stability constants for RTILs are taken from [21] for 25 °C, K_1_ = [Cs(18C6)][Cs]^−1^[18C6]^−1^ K_2_ = [Cs(18C6)_2_][Cs(18C6)]^−1^[18C6]^−1^; ^b^ Ref. [12]; ^c^ Ref. [9], pH = 7; ^d^ Ref. [9], C_o_^0^(18C6) = 1.0 × 10^−1^ mol·dm^-3^; ^e^ Ref. [9], C_o_^0^(18C6) = 1.0 × 10^−1^ mol·dm^-3^, blank experiment (without CsNO_3_); ^f^ Ref. [23]; ^g^ Ref. [2], picrate salt; ^h^ Ref. [3], picrate salt; ^i^ Ref. [1].

## References

[1] Takeda Y., Kawarabayashi A., Endō K., Yahata T., Kudo Y., Katsuta S. (1998). Solvent extraction of alkali metal (Li–Cs) picrates with 18-crown-6 into various diluents. Elucidation of fundamental equilibria which govern the extraction-ability and -selectivity. Anal. Sci..

[2] Levitskaia T.G., Maya L., van Berkel G.J., Moyer B.A. (2007). Anion Partitioning and ion-pairing behavior of anions in the extraction of cesium salts by 4,5‘‘-bis(*tert*-octylbenzo)dibenzo-24- crown-8 in 1,2-dichloroethane. Inorg. Chem..

[3] Abramov A.A. (2000). Extraction of cations by crown-ethers. Moscow Univ. Chem. Bull..

[4] Robak W., Apostoluk W., Maciejewski P. (2006). Analysis of liquid–liquid distribution constants of nonionizable crown ethers and their derivatives. Anal. Chim. Acta.

[5] Mohapatra P.K., Ansari S.A., Sarkar A., Bhattacharyya A., Manchanda V.K. (2006). Evaluation of calix-crown ionophores for selective separation of radio-cesium from acidic nuclear waste solution. Anal. Chim. Acta.

[6] Earle M.J., Seddon K.R. (2000). Ionic liquids. Green solvents for future. Pure Appl. Chem..

[7] Cocalia V.A., Holbrey J.D., Gutowski K.E., Bridges N.J., Rogers R.D. (2006). Separations of metal ions using ionic liquids: The challenges of multiple mechanisms. Tsinghua Sci. Technol..

[8] Dai S., Ju Y.H., Barnes C.E. (1999). Solvent extraction of strontium nitrate by a crown ether using room-temperature ionic liquids. J. Chem. Soc., Dalton Trans..

[9] Visser A.E., Swatloski R.P., Reichert W.M., Griffin S.T., Rogers R.D. (2000). Traditional extractants in nontraditional solvents: Groups 1 and 2 extraction by crown ethers in room-temperature ionic liquids. Ind. Eng. Chem. Res..

[10] Luo H., Dai S., Bonnesen P.V., Buchanan A.C. (2006). Separation of fission products based on ionic liquids: Task-specific ionic liquids containing an aza-crown ether fragment. J. Alloys Compounds.

[11] Chen P.Y. (2007). The assessment of removing strontium and cesium cations from aqueous solutions based on the combined methods of ionic liquid extraction and electrodeposition. Electrochim. Acta.

[12] Chun S., Dzyuba S.V., Bartsch R.A. (2001). Influence of structural variation in room-temperature ionic liquids on the selectivity and efficiency of competitive alkali metal salt extraction by a crown ether. Anal. Chem..

[13] Visser A.E., Rogers R.D. (2003). Room-temperature ionic liquids: New solvents for f-element separations and associated solution chemistry. J. Solid State Chem..

[14] Shimojo K., Goto M. (2004). Solvent Extraction and stripping of silver ions in room-temperature ionic liquids containing calixarenes. Anal. Chem..

[15] Visser A.E., Swatloski R.P., Reichert W.M., Mayton R., Sheff S., Wierzbicki A., Davis J.H., Rogers R.D. (2002). Task-Specific ionic liquids incorporating novel cations for the coordination and extraction of Hg^2+^ and Cd^2+^: synthesis, characterization, and extraction studies. Environ. Sci. Technol..

[16] Visser A.E., Swatloski R.P., Reichert W.M., Mayton R., Sheff S., Wierzbicki A., Davis J.H., Rogers R.D. (2001). Task-specific ionic liquids for the extraction of metal ions from aqueous solutions. Chem. Commun..

[17] Ajioka T., Oshima S., Hirayama N. (2008). Use of 8-sulfonamidoquinoline derivatives as chelate extraction reagents in ionic liquid extraction system. Talanta.

[18] Lohithakshan K.V., Aggarwal S.K. (2008). Solvent extraction studies of Pu(IV) with CMPO in 1-octyl- 3-methylimidazolium hexafluorophosphate (C_8_mimPF_6_) room temperature ionic liquid (RTIL). Radiochim. Acta.

[19] Dietz M.L., Stepinski D.C. (2008). Anion concentration-dependent partitioning mechanism in the extraction of uranium into room-temperature ionic liquids. Talanta.

[20] Nishi N., Murakami H., Imakura S., Kakiuchi T. (2006). Facilitated transfer of alkali-metal cations by dibenzo-18-crown-6 across the electrochemically polarized interface between an aqueous solution and a hydrophobic room-temperature ionic liquid. Anal. Chem..

[21] Vendilo A.G., Djigailo D.I., Rönkkömäki H., Lajunen M., Chernikova E.A., Lajunen L.H.J., Pletnev I.V., Popov K.I. (2010). Thermodynamics of cesium complexes formation with 18-crown-6 in hydrophobic ionic liquids. A correlation with extraction capability. Macrocyclic Chemistry: New Research Developments.

[22] Yakshin V.V., Vilkova O.N., Tsarenko N.A., Demin S.V., Zhilov V.I., Tsivadze A.Y. (2006). Molecular Design of macrocyclic extractants for extraction and separation of alkali and alkaline- earth metals. Russ. J. Coord. Chem..

[23] Kikuchi Y., Sakamoto Y. (2000). Complex formation of alkali metal ions with 18-crown-6 and its derivatives in 1,2-dichloroethane. Anal. Chim. Acta.

[24] Arnaud-Neu F., Delgado R., Chaves S. (2003). Critical evaluation of stability constants and thermodynamic functions of metal complexes of crown ethers. Pure Appl. Chem..

[25] IUPAC Stability Constants Database.

[26] Egorov V.M., Smirnova S.V., Pletnev I.V. (2008). Highly efficient extraction of phenols and aromatic amines into novel ionic liquids incorporating quaternary ammonium cation. Sep. Purif. Technol..

[27] Vilkova O.M., Yakshin V.V. (2003). Extraction-photometric determination of the composition of multicomponent crown ether—Podand mixtures. J. Anal. Chem..

